# Identification and validation of oxidative stress-related genes for the diagnosis of sepsis-induced acute lung injury

**DOI:** 10.1371/journal.pone.0327945

**Published:** 2025-07-22

**Authors:** Xue Fu, Jiawei Dong, Jian Yang, Xiaotian Zhang, Sen Wang, Shangkun Cai, Yiwei Zhang, Meng Zhang

**Affiliations:** 1 Department of Emergency, Hebei Medical University Third Hospital, Shijiazhuang, China; 2 Department of Thoracic Surgery, Hebei Medical University Third Hospital, Shijiazhuang, China; Jhargram Raj College, INDIA

## Abstract

Sepsis-induced acute lung injury (ALI) is an inflammatory pulmonary condition characterized by a complex pathophysiological mechanism. The development and progression of sepsis-induced ALI are accompanied by significant oxidative damage. This study aimed to identify key oxidative stress-related genes associated with sepsis-induced ALI. Samples, including sepsis, sepsis-induced ALI, and control groups, were obtained from the Gene Expression Omnibus database. Key oxidative stress-related genes in sepsis-induced ALI were identified using Weighted Gene Co-expression Network Analysis (WGCNA), Protein-Protein Interaction (PPI) network analysis, logistic regression, and LASSO regression analysis. Functional information regarding these genes was explored through Gene Set Variation Analysis (GSVA) and Gene Set Enrichment Analysis (GSEA). A logistic regression model was constructed based on the identified hub oxidative stress-related genes. The diagnostic value of this model for sepsis-induced ALI was assessed using the receiver operating characteristic (ROC) curve. The relative abundance of 22 human immune cell types was calculated using CIBERSORT software. The expression levels of hub genes in the blood samples of sepsis-induced ALI patients were analyzed through RT-PCR and ELISA. A total of 1,055 genes associated with sepsis-induced ALI were identified via WGCNA, of which 145 genes were linked to oxidative stress. GSVA revealed that these 145 genes were significantly enriched in 79 biological pathways, while GSEA indicated a strong association with immune-related signaling pathways. Additionally, the top 20 genes were selected through PPI network analysis. The logistic regression model was constructed using *VDAC1*, *HSPA8*, *SOD1*, *HSPA9*, *TXN*, and *SNCA*. In the training set and the validation set, the AUC values of logistic regression model were 0.9091 and 0.8279, respectively, suggesting good discriminability when distinguishing normal from sepsis-induced ALI. Notably, these six genes were correlated with immune cell infiltration in sepsis-induced ALI, with *HSPA8*, *SOD1*, and *HSPA9* showing downregulation in sepsis-induced ALI. In conclusion, *VDAC1*, *HSPA8*, *SOD1*, *HSPA9*, *TXN*, and *SNCA* have been identified as oxidative stress-related genes associated with sepsis-induced ALI. The logistic regression model developed using these six genes could identify patients with sepsis-induced ALI. Our findings might provide novel research strategies for the molecular therapeutic target of sepsis-induced ALI.

## 1. Introduction

Acute lung injury (ALI) is a prevalent critical illness characterized by pulmonary edema, oxidative stress, and inflammation, ultimately leading to impaired lung function [1,2]. ALI can arise from various factors, including sepsis, infection, trauma, shock, and blood transfusion [3]. Sepsis is the primary cause of ALI, accounting for approximately 40% of all cases [4]. Sepsis-induced ALI can progress into acute respiratory distress syndrome (ARDS), which may result in multiple organ dysfunction and death [5,6]. A study has found that 71 out of 160 patients (44%) developed ALI within 5 hours of septic shock onset, with ninety percent of patients experiencing ALI within the first 12 hours, of whom 64 (90%) met the ARDS criteria [7]. Currently, the primary treatment strategies for sepsis-induced ALI include pharmacological interventions and mechanical ventilation [8–11]. However, while these treatments may alleviate clinical symptoms, they do not significantly improve the prognosis of patients with sepsis-induced ALI, with the mortality rate remaining as high as 46% [12]. Therefore, further exploration of the effective biomarkers and treatment strategies is essential to improve the cure rate and reduce the mortality rate of sepsis-induced ALI.

Oxidative stress is defined as an imbalance between the production of reactive oxygen species (ROS) and the neutralizing capacity of the cell’s redox buffering system, particularly involving glutathione [13]. In sepsis-induced ALI patients, the oxidants generated by activated lung macrophages or oxidant-generating enzymes entering the lung could exacerbate pulmonary inflammation and tissue damage [14]. In addition, the level of peroxynitrite (a potent oxidant) is increased in ALI patients [15]. Collectively, reducing the oxidative stress could ameliorate the ALI. Ye et al have reported that the overexpression of *MUC1* could increase the superoxide dismutase (SOD) level and catalase (CAT) and glutathione catalase (GSH-Px) activities and decrease the myeloperoxidase (MPO) activity and malondialdehyde (MDA) content in lipopolysaccharide (LPS)-induced ALI cells [16]. Gly-Pro-Ala protein could reduce the ROS level and improve SOD activity in alveolar macrophage, thereby relieving the sepsis-induced ALI in mice [17]. Accordingly, oxidative stress is involved in the pathogenesis of sepsis-induced ALI and may provide new strategies for the treatment of sepsis-induced ALI by regulating oxidative stress-related genes and proteins.

Currently, some studies have indicated that gene expression signatures have great predictive value to identify sepsis induced ALI patients [18]. For example, one study found that a signature of five genes is associated with ALI and can be used to distinguish patients with sepsis-induced ALI from patients with sepsis [19]. However, to the best of our knowledge, there are few reports that have investigated the role of oxidative stress-related gene signature in sepsis-induced ALI. Machine learning is an emerging field capable of handling large-scale, complex, and diverse datasets. This technology has significantly improved our ability to identify relevant features from gene expression profiles, particularly when addressing high-dimensional data [20]. Machine learning method has the potential to identify biomarkers and aid in the diagnosis of many diseases [21,22]. Therefore, in this work, we identified the oxidative stress-related genes in sepsis-induced ALI by collecting and analyzing the data from the Gene Expression Omnibus (GEO) database via integrating multiple machine learning algorithms. Our reports potentially provide a novel target for the development of the diagnostic strategies for sepsis-induced ALI.

## 2. Methods

### 2.1. Data source

The GSE32707, GSE10361, GSE3037, GSE172222, and GSE66890 datasets were obtained from GEO database (https://www.ncbi.nlm.nih.gov/geo/). The GSE32707 dataset included 58 whole blood samples from sepsis patients, 52 whole blood samples from sepsis-induced ALI patients, and 34 control whole blood samples. Data for this dataset were obtained using the Illumina HumanHT-12 V4.0 expression beadchip (GPL10558) platform. The GSE10361 dataset comprised 6 whole blood samples from sepsis-induced ALI patients and 3 control whole blood samples, with data collected using the Affymetrix Human Genome U133A Array (GPL96) platform. The GSE3037 dataset included 16 peripheral blood samples from sepsis-induced ALI patients and 8 control peripheral blood samples, also obtained from the Affymetrix Human Genome U133A Array (GPL96) platform. Lastly, the GSE17222 dataset contained 31 lung epithelial cell samples from sepsis-induced ALI patients and 8 control lung epithelial cell samples, with data sourced from the Affymetrix Human Gene 2.0 ST Array (GPL20103) platform. Moreover, the GSE66890 dataset included 28 sepsis and 29 sepsis-induced ARDS (whole blood), with data sourced from the Affymetrix Human Gene 1.0 ST Array (GPL6244) platform. The missing values were filled with the mean. The datasets GSE32707 and GSE10361 were merged and batch effects were removed to create the meta-GEO cohort (included 58 sepsis, 52 sepsis-induced ALI, and 37 control samples), which served as the training set. Similarly, the GSE3037 and GSE172222 datasets were combined and batch effects were removed to form training set (included 48 sepsis-induced ALI samples and 16 control samples). The R packages “limma” and “sva” were used to mitigate batch effects. The sample mean and standard deviation were calculated, and then the effect size (Cohen’s d) of the dataset sample was calculated using the ‘pwr’ package in R to ensure that the study had sufficient power to detect meaningful effects.

### 2.2. Weighted gene co-expression network analysis (WGCNA) WGCNA

Based on the expression values of genes, the top 25% of genes were screened by variance analysis to WGCNA using the “WGCNA” package [23] in R language (version 4.2.1, the same below). Next, the Kolmogorov-Smirnov test was used to determine if variables followed a normal distribution. Pearson correlation analysis was conducted for the measurement items conforming to a normal distribution (S1 Table, *p*  <  0.05), and selected appropriate soft thresholds β. The one-step method was applied to build a gene network, the adjacency matrix was transformed into a topological overlap matrix (TOM), and the hierarchical clustering was applied to produce a hierarchical clustering tree. The significances of genes and modules were calculated to measure the significance between genes and clinical information, and the significant association between modules and traits was analyzed to obtain gene modules.

### 2.3. Function analysis

To explore the biological pathways of sepsis-induced ALI-related oxidative stress genes, the Gene set variation analysis (GSVA) was preformed using by “GSVA” in R [24]. GSVA is a non-parametric and unsupervised method for assessing the alterations in the biological pathways and processes utilizing the gene expression values. The standard gene sets were constructed using the “c2.cp.kegg.v2022.1.Hs.symbolsgene sets from the Molecular signatures database (https://www.gsea-msigdb.org/gsea/msigdb). To gain a deeper understanding of the potential mechanisms underlying oxidative stress-related genes in sepsis-induced ALI, we analyzed the expression of sepsis-induced ALI-related oxidative stress genes in both sepsis-induced ALI and control groups. GSEA was performed to determine whether the differentially expressed oxidative stress- related genes in the two groups were significantly enriched in any biological processes or pathways using the “ClusterProfiler” [25] packages in R. The significantly enriched pathways were identified by adjust *p* < 0.05 (Benjamini–Hochberg (BH) method).

### 2.4. Protein–protein interaction (PPI) network analysis

The STRING (https://string-db.org/,version 11.0) database [26] was employed to analyze the functional interactions between proteins. The Cytoscape (version 3.7.2) [27] was applied to visualize the PPI network. Based on the maximum neighborhood component (MNC) algorithm, the Cytohubba plugin in Cytoscape was applied to further screen the hub genes in the PPI network.

### 2.5. Construction of Logistic regression model β

Logistic regression is a widely utilized method for classification that predicts outcomes based on a set of variables. In this study, logistic regression was employed to determine whether the samples represented sepsis-induced ALI. Specifically, a multiple logistic regression model was constructed using the ‘glm’ function in R, incorporating gene expression levels as continuous independent variables and sample types (ALI samples versus control samples) as dichotomous response values. Independent variables included in the model were selected through stepwise regression using the ‘step’ function in R, with a significance threshold of *p*  <  0.05. Variants with *p* < 0.05 were identified as candidate genes, and the parameter ‘family=binomial(link=“logit”)’ was applied to reconstruct the final model for subsequent analyses. Multicollinearity among the variables was tested using the variance inflation factor (VIF). The stability and accuracy of the model were evaluated using receiver operating characteristic (ROC) curves and assessed by area under the curve (AUC) values. An AUC of 0.5–0.7 indicated low predictive value; AUC of 0.7–0.9 indicated moderate predictive value; and AUC > 0.9 indicated high predictive value.

### 2.6. Immune cell infiltration

The relative contents of 22 immune cells were calculated by CIBERSORT software [28]. In the CIBERSORT analysis, the permutation number was set to perm = 100 and the quantile normalization (QN) was set to QN = TRUE. According to the gene expression matrix, CIBERSORT can describe the composition of immune infiltrating cells using the 547 preset barcode genes in the deconvolution algorithm. The sum of all estimated immune cell type proportions in each sample equals 1. The differences in immune cell infiltration between groups were compared by the Wilcoxon rank sum test. The developers of CIBERSORT initially designed and validated a leukocyte gene signature set, LM22, which comprises 574 genes capable of distinguishing 22 hematopoietic cell phenotypes. These include seven types of T cells, naive and memory B cells, plasma cells, natural killer (NK) cells, and various myeloid subsets. Utilizing LM22, the relative proportions of these 22 immune cell types are calculated through the deconvolution of tissue RNA sequencing data. CIBERSORT performs deconvolution through the formula: Y = X × β + ε. Y: Gene expression matrix of mixed samples (m × nm × n, where m is the number of genes and n is the number of samples); X: Reference feature matrix (m × km × k, k = 22 immune cell types); β: The cell proportionality coefficient matrix (k × nk × n) to be solved; E: Error term.

Moreover, xCell [29] (https://github.com/dviraran/xCell) can calculate the abundance of 64 cell types in the tumor microenvironment based on tissue transcriptome data using a combination of single-sample gene set enrichment analysis (ssGSEA) and deconvolution methods. The 64 cell types include myeloid cells (13 types), lymphoid cells (21 types), stem cells (9 types), stromal cells (13 types), and other cell types (8 types).

### 2.7. Clinical sample collection

Whole blood samples were collected from 20 patients with sepsis-induced ALI and 20 healthy individuals at the Third Hospital of Hebei Medical University between September 18, 2024, and October 4, 2024. Informed consent was obtained from each participant, who signed a consent form prior to the study. The research protocol received approval from the Ethics Committee of the Third Hospital of Hebei Medical University (Approval No. KS2024-236-1). The clinical data of the subjects are summarized in Table 1.

**Table 1:** Clinical information of sepsis-induced acute lung injury and healthy individuals.

Clinical characteristics	Sepsis-induced-ALI (N = 20)	Healthy (N = 20)	P value
Age (years)	60.55 ± 14.92	33.05 ± 9.74	0.00057
Female, n (%)	4 (20%)	12 (60%)	/
Body mass index	23.96 ± 3.01	25.30 ± 4.35	0.073
White cell count on admission (x10^9^)	11.49 ± 4.62		/
SOFA score on admission, median (IQR)	8.70 ± 4.33		/
APACHE II score on admission	19.50 ± 5.05		/

### 2.8. RT-qPCR assay

The extraction of total RNA from whole blood was carried out using the RNAprep Pure High Efficiency Blood Total RNA Extraction Kit (DP443, Tiangen Biotech, Co., Ltd., Beijing, China). For reverse transcription, the Evo M-MLV Reverse Transcription Premix Kit (AG11728, Accurate Biology, Changsha, China) was utilized. Following this, the qRT-PCR assay was executed using the SuperStar Universal SYBR Master Mix (CW3360M, Jiangsu Cowin Biotech Co., Ltd., Jiangsu, China) on a real-time fluorescence quantitative PCR instrument (SLAN-96S, Shanghai Hongshi Medical Technology Co., Ltd, Shanghai, China). The cycling program included a pre-denaturation step at 95°C for 30 seconds, followed by 40 cycles of 95°C for 10 seconds and 60°C for 30 seconds. GADPH served as the internal control. The expression levels of mRNA were quantified using the 2^-ΔΔCT^ method. The primer sequences for RT-qPCR are as follows:

#### VDAC1

: 5′ – AAACAAAATCTGAGAATGGATTGGA – 3′ (F); 5′ – AGCCCAGGTTAATGTGCTCC – 3′ (R)

##### HSPA8

: 5′ – CAGGTTTATGAAGGCGAGCG – 3′ (F); 5′ - CGTACTCTTGTCCACAGCAGA −3′ (R)

##### HSPA9

: 5′ – AGAAGACCGGCGAAAGAAGG – 3′ (F); 5′ – GCCAGGAGCTCCCTCATTTT − 3′ (R)

##### GAPDH

: 5′ – GAAGGTGAAGGTCGGAGTC – 3′ (F); 5′ – GAAGATGGTGATGGGATTTC − 3′ (R).

### 2.9. Enzyme linked immunosorbent assay (ELISA)

Whole blood was collected into a test tube without anticoagulant, and then centrifuged at approximately 1000 × g for 10 minutes at 4°C. The yellow supernatant was carefully extracted to obtain serum. The concentrations of HSPA8 and HSPA9 in the serum were specifically measured using the human HSPA8 ELISA KIT (ml062917V, Enzyme-Linked Biology, Shanghai, China) and the Human HSP-70 ELISA KIT (ml963761V, Enzyme-Linked Biology, Shanghai, China), respectively, following the manufacturer’s instructions. The optical density (OD) value was determined using a microplate reader at a wavelength of 450 nm.

## 3. Results

### 3.1. Identification of sepsis-induced ALI onset-related genes

**Fig 1:**
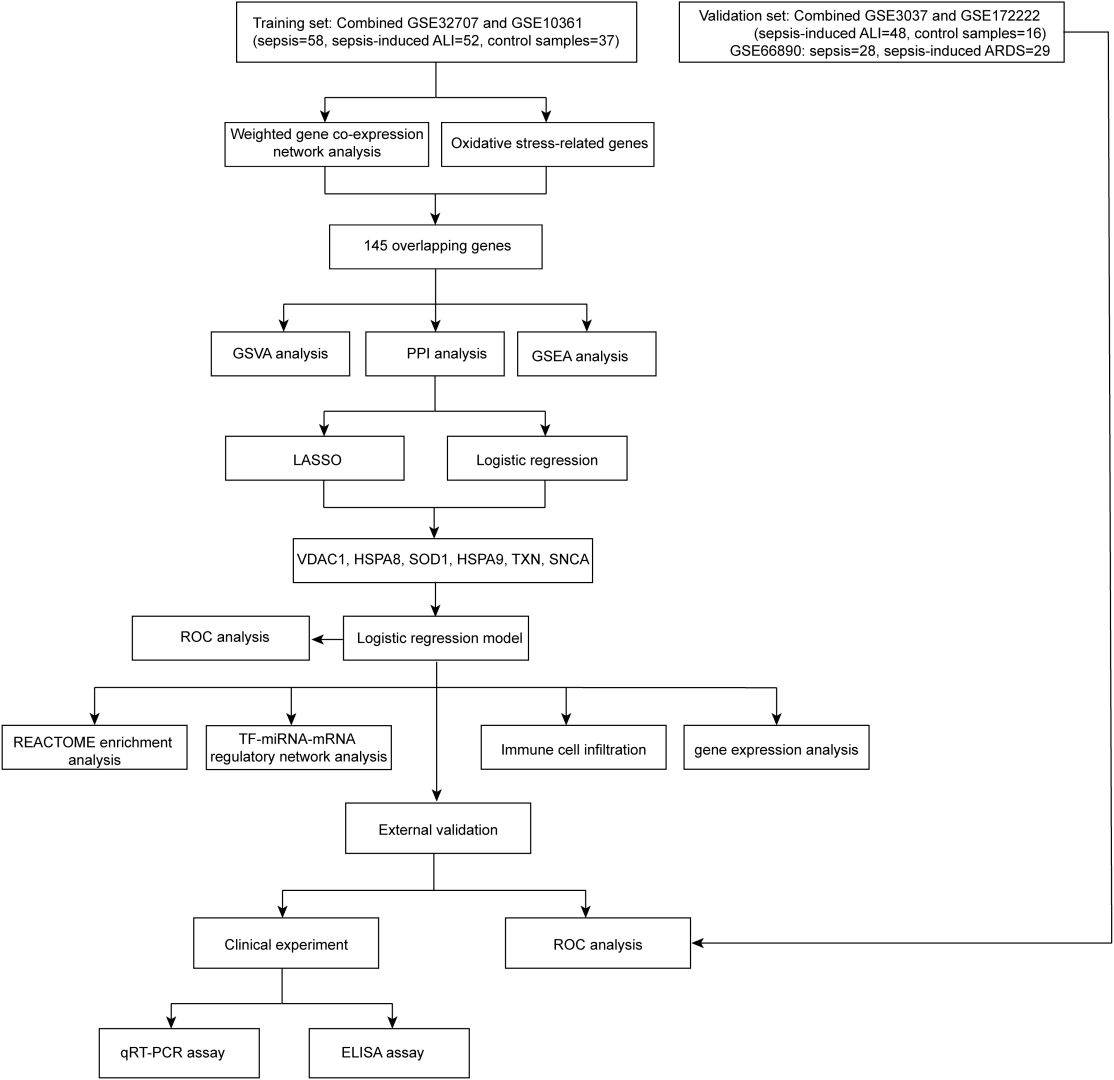
The workflow of this article.

**Fig 2:**
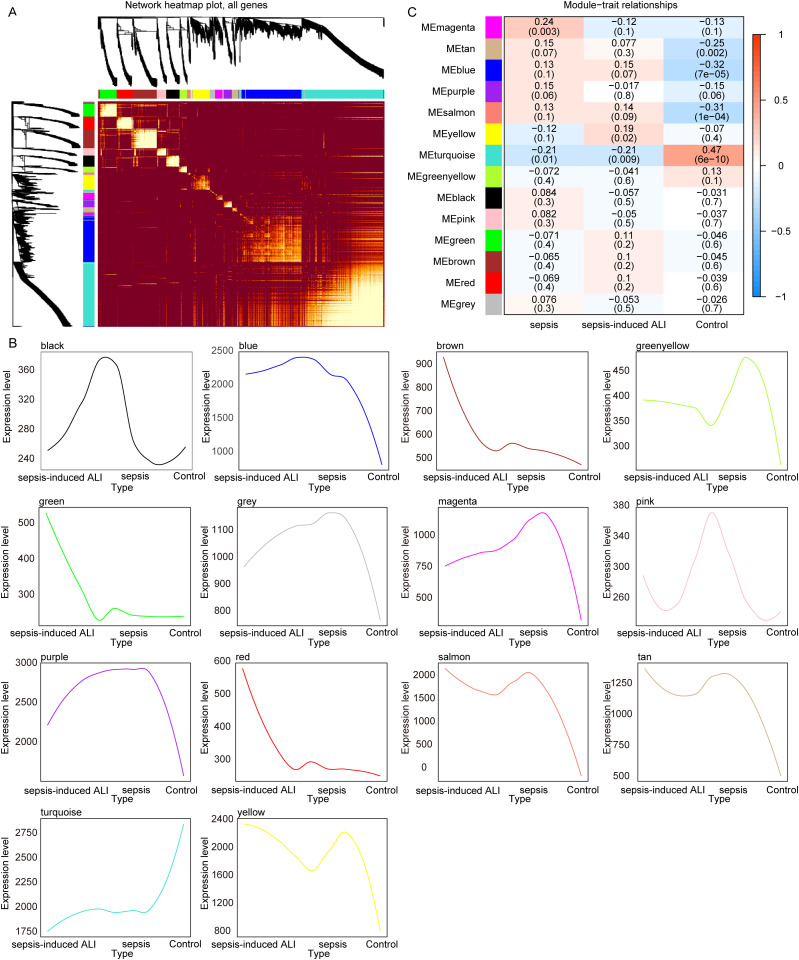
Identification of sepsis-induced ALI onset related genes. A. The heat map of module genes. B. The expression level of genes in each module. C. The correlation of gene modules with sepsis-induced ALI. The color bar on the far right illustrates the range of correlation. Red indicates a positive correlation, and blue signifies a negative correlation. Darker shades indicating stronger correlations. Each number in the cells denotes the correlation value and its significance.

The sample types in the GSE32707 and GSE10361 datasets are identical, as both consist of whole blood samples from sepsis, sepsis-induced ALI and control, so we merged these two datasets. The R packages “limma” and “sva” were utilized to mitigate batch effects (S1 Fig), thereby eliminating experimental errors attributed to variations in experimental times, operators, reagents, and instruments. The merged dataset consists of 58 sepsis, 52 sepsis-induced ALI, and 37 control samples that we named a meta-GEO cohort (Cohen’s d = 0.442). A flowchart summarizing results of this article selection process is shown in Fig 1. To identify gene modules associated with the onset of sepsis-induced ALI, we constructed a WGCNA using samples from the meta-GEO cohort. We determined β = 7 (S2 Fig) as the optimal soft threshold, resulting in the identification of 14 gene modules (Fig 2A) for subsequent analysis. The expression trends of the genes within each module are illustrated in Fig 2B. Among these 14 gene modules, two (yellow and turquoise) exhibited significant associations with sepsis-induced ALI (Fig 2C). A total of 1,055 genes were included in the yellow and turquoise modules (S2 Table), which were designated as sepsis-induced ALI-related genes for further analysis.

To clarify the potential function of these 1,055 genes in sepsis-induced ALI, these genes were analyzed using the ClueGO plugin of Cytoscape software. As shown in S3 Fig, these genes were significantly enriched in 19 KEGG pathways (S3A Fig), 35 GO_BP pathways (S3B Fig), 28 GO_CC pathways (S3C Fig), and 18 GO_MF pathways (S3D Fig). The detailed results were shown in S3 Table.

### 3.2. Identification of oxidative stress-related target genes in sepsis-induced ALI

**Fig 3:**
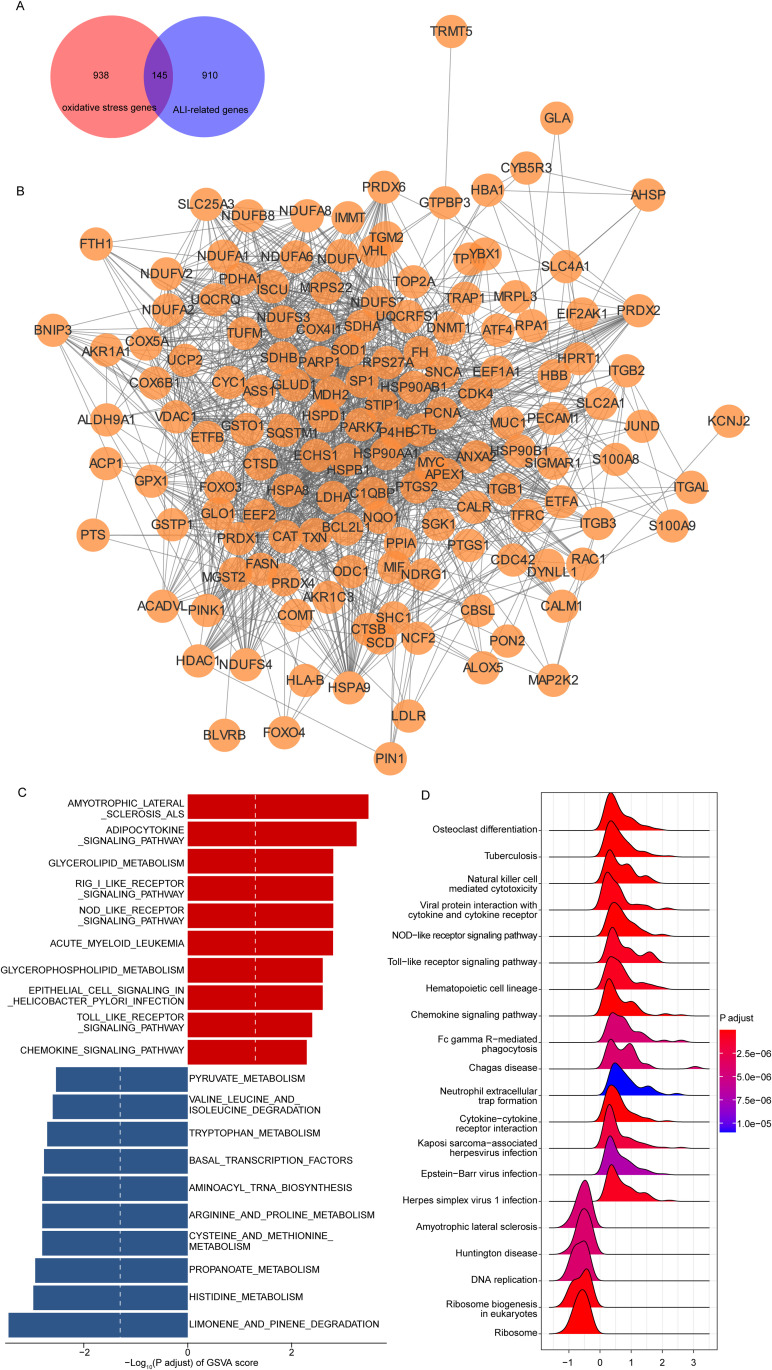
Identification of oxidative stress-related target genes in sepsis-induced ALI. A. The overlapping genes between sepsis-induced ALI-related genes group and oxidative stress-related genes group. B. The result of the PPI network. The enriched pathways of 145 sepsis onset-related genes using GSVA (C) and GSEA (D).

Previous studies have demonstrated that oxidative stress plays a critical role in sepsis-induced ALI, and that inhibiting excessive ROS may improve pulmonary function and survival outcomes [30]. Consequently, we next identified oxidative stress-related genes associated with the pathogenesis of sepsis-induced ALI. First, Taking “oxidative stress” as a keyword, we identified a total of 1,083 oxidative stress-related genes from GeneCards (https://www.genecards.org) based on a relevance score of ≥7. Furthermore, we conducted a cross-over analysis between 1,055 sepsis-induced ALI-related genes and 1,083 oxidative stress-related genes, resulting in the identification of 145 overlapping genes (Fig 3A). These 145 genes were listed in S4 Table. Subsequently, we established a PPI network based on these 145 genes using the STRING database, a threshold of minimum required interaction score > 0.4 was used to screen interaction pairs (Fig 3B).

### 3.3. Functional enrichment analysis of sepsis-induced ALI-related oxidative stress genes

To analyze the potential pathways affected by 145 sepsis-induced ALI-related oxidative stress gene expression, we performed a GSVA in the meta-GEO cohort. As shown in Fig 3C, these 145 genes were significantly enriched in 79 pathways by GSVA analysis (adjust *p* < 0.05), such as amyotrophic lateral sclerosis ALS, adipocytokine signaling pathway, propanoate metabolism, histidine-metabolism and limonene and pinene degradation pathways. Furthermore, to gain a deeper understanding of the potential mechanisms of 145 sepsis-induced ALI-related oxidative stress gene in sepsis-induced ALI, the GSEA were performed. The GSEA results showed that there 145 genes were closely associated with immune related signaling pathways, such as natural killer cell mediated cytotoxicity, NOD-like receptor signaling pathway, and Toll-like receptor signaling pathway (Fig 3D, adjust *p* < 0.05). Detailed results of the GSVA and GSEA are presented in S5 Table.

### 3.4. Construction of a logistic regression model for sepsis-induced ALI using *VDAC1*, *HSPA8*, *SOD1*, *HSPA9*, *TXN* and *SNCA*

**Fig 4:**
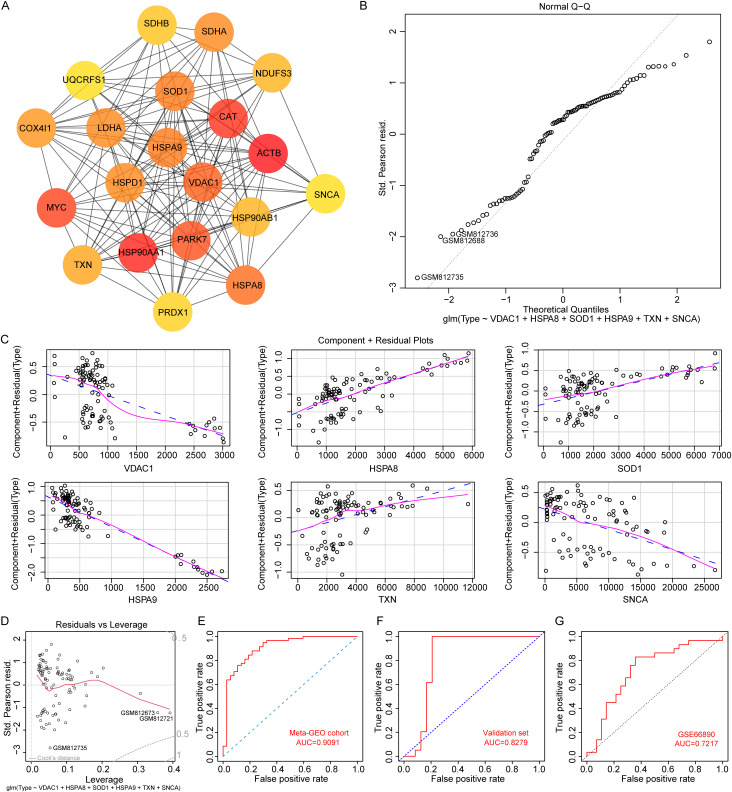
Diagnostic model construction for sepsis-induced ALI. A. The top 20 genes in the PPI network. B. Normality of residuals was confirmed through QQ plot of residuals. The normal QQ plot can be used to test whether a data sequence conforms to a certain probability distribution. It creates a scatter plot with the probability quantiles of the corresponding distribution as the horizontal axis and the quantiles of the data sequence as the vertical axis. C. The component residual plots for the 6 genes in the logistic regression model. The presence of a noticeable linear relationship between the horizontal and vertical axes in the graph suggests that the independent variable is appropriate for inclusion in the model. D. The leverage plot of residual. The ROC curve of the model in meta-GEO cohort (E), validation set (F), and GSE66890 dataset (G).

To further screen for oxidative stress-related genes associated with the pathogenesis of sepsis-induced ALI, we assessed the significance of each node within the network using the MNC algorithm based on the PPI network analysis. Nodes with darker colors indicated higher importance. The top 20 genes were then identified based on their scores, ranked from highest to lowest (Fig 4A, S6 Table). Subsequently, we employed two machine learning algorithms, LASSO and logistic regression, to further screen for hub genes. Using the LASSO regression algorithm, a total of 9 genes (*TXN*, *SNCA*, *ACTB*, *MYC*, *PARK7*, *VDAC1*, *HSPA8*, *SOD1*, *HSPA9*) were selected from these 20 genes (S4 Fig). The logistic regression algorithm identified 8 genes (*VDAC1*, *HSPA8*, *SOD1*, *HSPA9*, *TXN*, *NDUFS3*, *HSP90AB1*, *SNCA*), among which 6 genes were closely correlated with the onset of sepsis-induced ALI (S7 Table, *p* < 0.05). By taking the intersection of the genes obtained using the two algorithms, a total of 6 hub oxidative stress-related genes were obtained, namely (*VDAC1*, *HSPA8*, *SOD1*, *HSPA9*, *TXN*, *SNCA*). Finally, these 6 genes were included as variables in the model, and the model evaluation showed a normal distribution (Fig 4B). These six genes were used as input genes and fed into the model. There was a good linear relationship between the independent variables and the response variable in the model (Fig 4C), and there was no significant extreme point affecting the accuracy of the model (Fig 4D). We constructed a confusion matrix and calculated the model’s Sensitivity, Specificity, Accuracy, Positive Predictive Value (PPV), and Negative Predictive Value (NPV). Our calculations yielded the following results: Sensitivity = 0.8793, Specificity = 0.7568, Accuracy = 0.8316, PPV = 0.85, and NPV = 0.8 (S8 Table). The area under the curve (AUC) of this model reached 0.9091 in the meta-GEO cohort (Fig 4E). This high AUC value indicates that the diagnostic model exhibits excellent accuracy for sepsis-induced ALI, with values approaching 1.0 representing near-perfect discrimination between disease and non-disease states. However, this result requires independent queues with larger sample sizes to verify, so we merged the samples in the GSE3037 and GSE172222 datasets. The R packages “limma” and “sva” packages were used to remove differences between batches (S5 Fig), experimental errors caused by different experimental times, operators, reagents, and instruments were eliminated. The merged dataset consists of 48 sepsis-induced ALI samples and 16 control samples that we named the validation set (Cohen’s d = 0.54). As shown in Fig 4F, the AUC of model was 0.8279 in the validation set. Furthermore, we downloaded the GSE66890 dataset from the GEO database, which includes 28 sepsis and 29 sepsis-induced ARDS. Subsequently, we conducted a ROC analysis based on an oxidative stress-related diagnostic model, yielding an AUC of 0.7217 (Fig 4G). This result suggests that the oxidative stress-related diagnostic model may effectively differentiate between sepsis-induced ARDS and sepsis.

### 3.5 Pathway enrichment analysis

**Fig 5:**
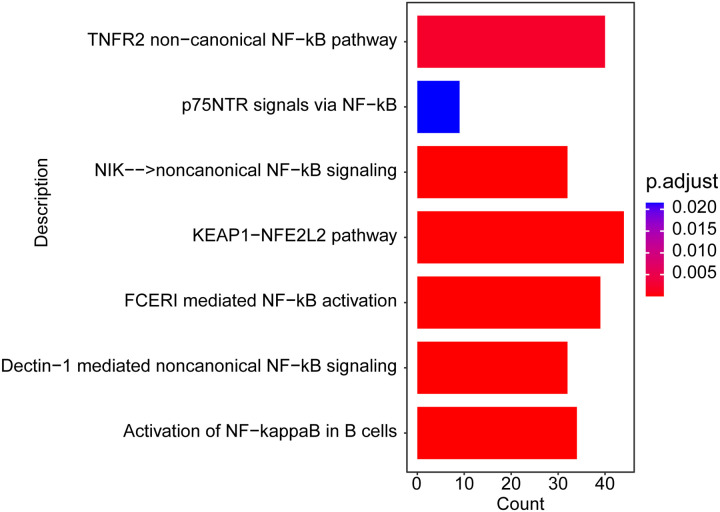
Top 7 significantly enriched Reactome pathway.

In the meta-GEO cohort, we used the expression of these 6 genes to divide the sepsis-induced ALI samples into high expression groups and low expression groups and perform differential expression analysis. Besides, a pathway enrichment analysis of the DEGs was performed using the pathways from Reactome database. As shown in Fig 5 and S9 Table, the DEGs were significantly enriched in oxidative stress related pathways, such as NF − κB signaling pathway and KEAP1-NRF2/NFE2L2 pathway (adjust *p* < 0.05).

### 3.6 TF-miRNA-mRNA regulatory network analysis

**Fig 6:**
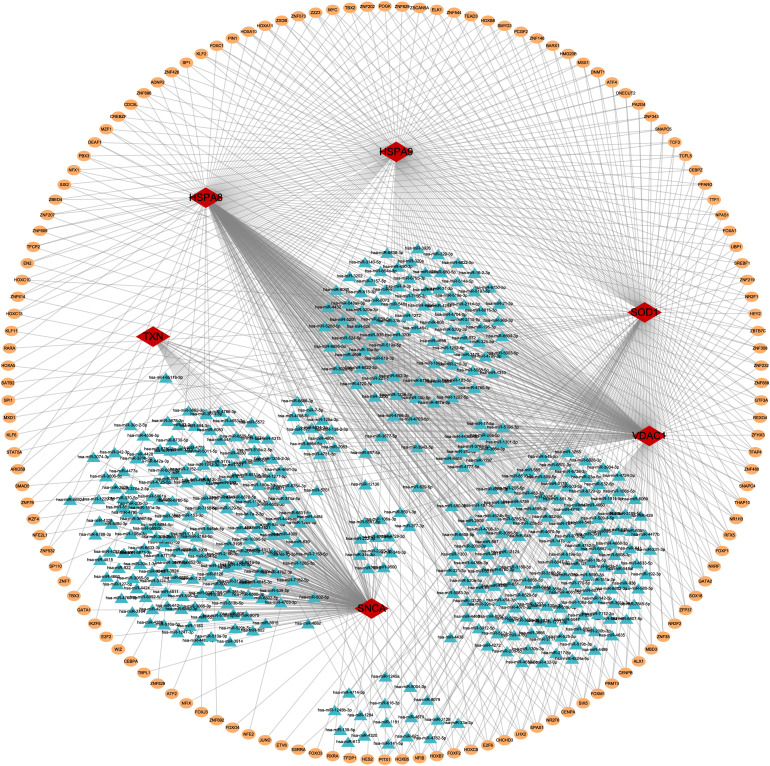
The regulatory network of TF-miRNA-mRNA. Red diamonds represent model genes, blue triangles represent miRNAs, and yellow circles represent TFs.

To explore the regulatory mechanisms of 6 genes in sepsis-induced ALI, we first collected upstream transcription factors (TFs) from the Human Transcription Factors Database. We calculated the correlation of TFs for *VDAC1*, *HSPA8*, *SOD1*, *HSPA9*, *TXN,* and *SNCA* in the meta-GEO cohort. Based on a correlation coefficient greater than 0.6 and a *p*-value less than 0.05, we identified TFs that were potentially associated with the expression of these six genes (S10 Table). Subsequently, we predicted the targeted miRNAs of model genes using TargetScan. Meanwhile, the top 5% of miRNAs with potential interactions with the model genes were selected using the mirDIP database. The selected TFs, miRNAs, and model genes were visualized in a network using Cytoscape (Fig 6).

**3.7 Correlation of *VDAC1*, *HSPA8*, *SOD1*, *HSPA9*, *TXN*, and *SNCA* with immune cell infiltration in sepsis-induced ALI**

**Fig 7:**
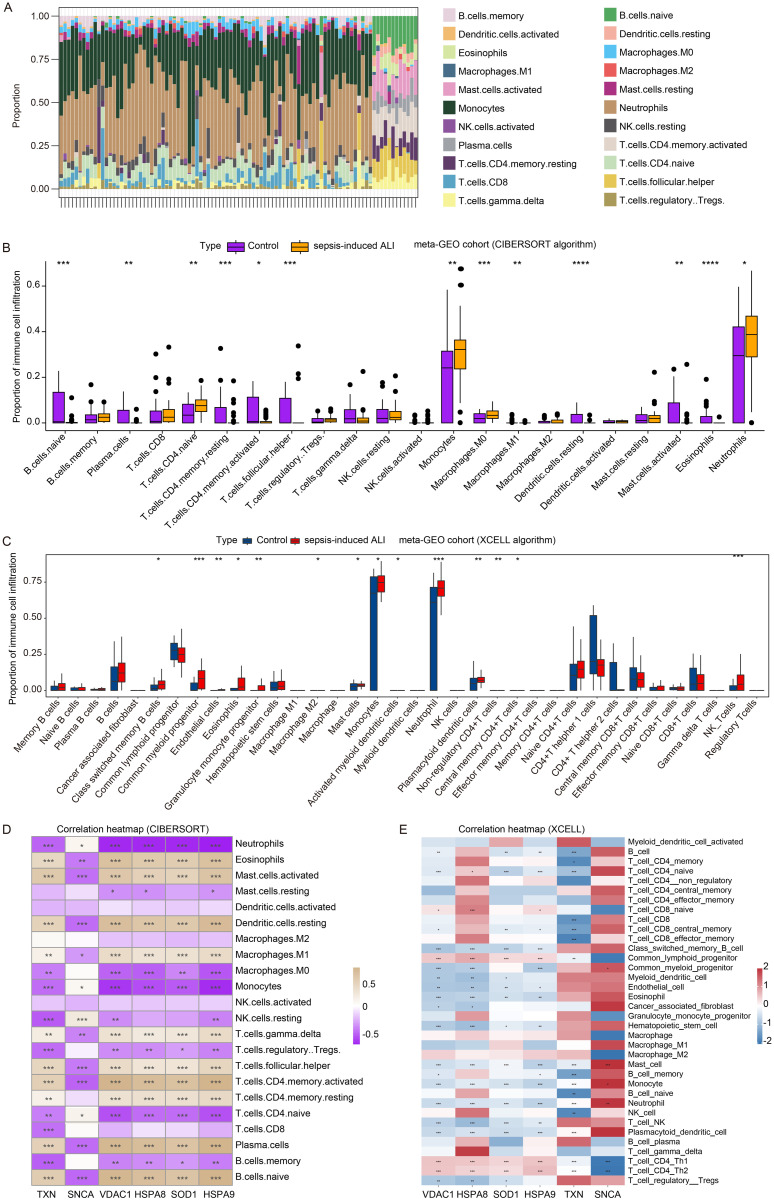
Correlation of *VDAC1*, *HSPA8*, *SOD1*, *HSPA9*, *TXN*, and *SNCA* with immune cell infiltration in sepsis-induced ALI. (A) The relative contents of 22 immune cell infiltration of samples in the meta-GEO cohort. The proportion of immune cell infiltration in the sepsis-induced ALI and control samples in the meta-GEO cohort calculated by CIBERSORT (B) and XCELL algorithms **(C)**. The correlation between the proportion of immune cell infiltration and *VDAC1*, *HSPA8*, *SOD1*, *HSPA9*, *TXN* and *SNCA* expression calculated by CIBERSORT (D) and XCELL algorithms **(E)**. *p < 0.05, **p < 0.01, ***p < 0.001, ****p < 0.0001.

To investigate the relationship between the model genes and the immune of sepsis-induced ALI, we calculated the different immune cell infiltration scores of samples in the meta-GEO cohort (52 sepsis-induced ALI samples and 37 control samples) using the CIBERSORT and XCELL algorithms. As shown in Fig 7A and B, naive B cells, plasma cells, resting memory CD4 + T cells, activated memory CD4 + T cells, follicular helper T cells, macrophages M1, resting dendritic cells, activated mast cells, eosinophils were significantly reduced in sepsis-induced ALI samples, and naive CD4 + T cells, Monocytes, macrophages.M0, and Neutrophils were increased. Furthermore, the Xcell algorithm was employed to visualize immune cell infiltration, revealing that the proportions of mast cells, monocytes and neutrophils were significantly elevated in sepsis-induced ALI (Fig 7C). Furthermore, the correlation coefficients landscape calculated by CIBERSORT (Fig 7D) and XCELL (Fig 7E) algorithms showed that the proportion of monocytes exhibited a significant negative correlation with *VDAC1, HSPA8, SOD1,* and *HSPA9* expressions, and a significant positive correlation with *SNCA* expression. Moreover, the proportion of neutrophils was significantly negatively associated with *VDAC1, HSPA8, SOD1, HSPA9* and *TXN* expressions and was positively correlated with *SNCA* expression (Fig 7D and E). These results indicated that in sepsis-induced ALI, when neutrophils are overactivated and release excessive ROS, the expression of oxidative stress – related genes may decrease as a compensatory mechanism to alleviate oxidative stress.

### 3.8. Expression of *HSPA8*, *SOD1*, and *HSPA9* in sepsis-induced ALI

**Fig 8:**
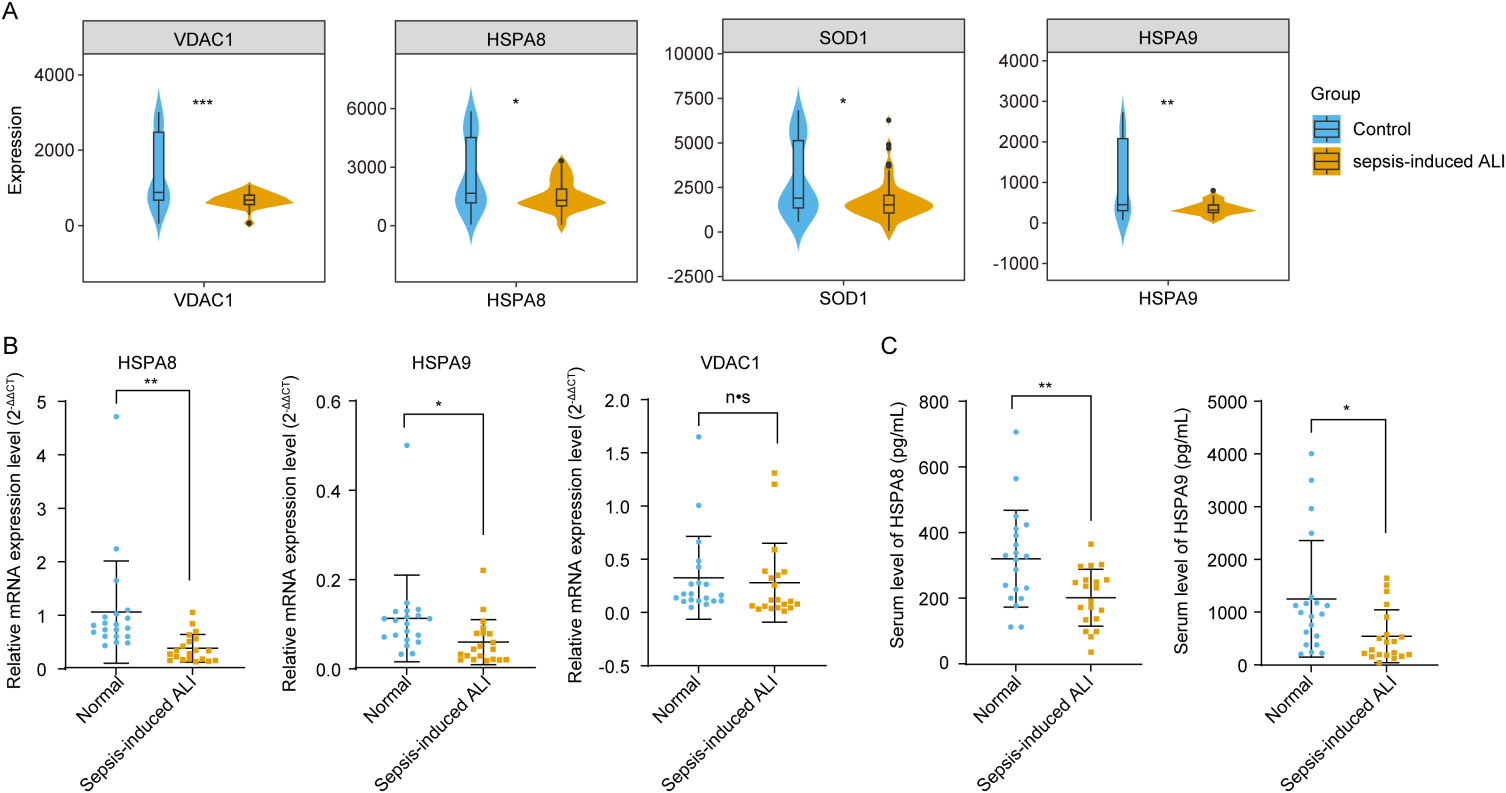
The expression of hub gene expression in sepsis-induced ALI. A. The expression of *VDAC1, HSPA8, SOD1, HSPA9* in sepsis-induced ALI in the meta-GEO cohort. B. The level of *HSPA8* and *HSPA9* miRNA expression in the blood samples of patients with sepsis-induced ALI. C. The concentration of *HSPA8* and *HSPA9* protein in the serum of patients with sepsis-induced ALI. *p < 0.05, **p < 0.01.

Finally, we analyzed the expression of *VDAC1, HSPA8, SOD1, HSPA9, TXN*, and *SNCA* in sepsis-induced ALI in the meta-GEO cohort. As shown in Fig 8A, the expression levels of *VDAC1*, *HSPA8*, *SOD1*, and *HSPA9* were significantly decreased in the sepsis-induced ALI group compared to the control group (*p* < 0.0.5). Previous studies have demonstrated that Sod1 is significantly reduced in a mouse model of sepsis-induced ALI [31]. Therefore, we next analyzed the expression of *VDAC1*, *HSPA8* and *HSPA9* in the blood samples of patients with sepsis-induced ALI at the clinical level. Our findings revealed that both the mRNA expression levels and protein concentrations of *HSPA8* and *HSPA9* were significantly reduced in the blood samples of patients suffering from sepsis-induced ALI (Fig 8B, C).

## 4. Discussion

In sepsis-induced ALI, the activation of inflammatory cells and the release of inflammatory mediators can induce oxidative stress, resulting in alveolar epithelial dysfunction [32]. The clinical diagnosis of sepsis-induced ALI primarily relies on the PaO2/FiO2 ratio and chest imaging, often neglecting molecular biological characteristics. This limitation hinders the timely provision of optimal clinical care for patients suffering from sepsis [33,34]. In this study, we constructed a logistic regression model constructed utilizing 6 oxidative stress-related genes, which demonstrated significant diagnostic value for sepsis-induced ALI.

The study has identified 6 oxidative stress-related genes (*VDAC1*, *HSPA8*, *HSPA9*, *SOD1*, *TXN*, and *SNCA*) that was closely correlated with the onset of sepsis-induced ALI through several machine learning algorithms *VDAC1* is located on the outer membrane of mitochondria and serves as a crucial channel that facilitates the transport of metabolites and ions in and out of these organelles [35]. As a mitochondrial gatekeeper, *VDAC1* is integral to the regulation of ROS release, mitochondrial metabolism, and energy production, thereby playing a crucial role in apoptosis mediated by mitochondria [36]. Oxidative stress is one of the primary pathological factors contributing to sepsis-induced ALI. Disruption of *VDAC1* oligomerization leads to altered intracellular Ca^2+^ concentrations and reduced ROS levels, thereby protecting against mitochondrial dysfunction induced by inflammation and apoptosis [37,38]. These findings further support that Cai et al. have found that the knockdown of *VDAC1* could decrease the autophagy and enhance efferocytosis, resulting in alleviated cognitive dysfunction in caecal ligation and puncture (CLP)-induced sepsis-associated encephalopathy [39]. Meanwhile, *TMBIM6* overexpression has been shown to enhance the interaction with *VDAC1*, thereby protecting myocardial mitochondria and cardiac function against sepsis [40]. This suggests that *VDAC1* may mitigate sepsis-induced organ damage by regulating mitochondrial dysfunction and oxidative stress. However, in contrast to previous, this study found that there was no significant difference in the expression of *VDAC1* in serum from patients with sepsis-induced ALI and healthy individuals. This observation may be attributed to the fact that *VDAC1* is primarily localized to the outer mitochondrial membrane, where it functions as a crucial channel for the transport of mitochondrial metabolites and ions [41]. Its role is predominantly associated with intracellular mitochondrial metabolic and signaling processes, rather than being directly secreted into the extracellular space [41]. Consequently, the serum levels of *VDAC1* may not accurately reflect its functional status within the cells.

Heat shock protein member 8 (*HSPA8*) and heat shock protein member 9 (*HSPA9*) are members of the heat shock protein 70 (HSP70) family [42]. *HSPA8* is one of the most abundantly expressed chaperones in the cytosol and nucleus [42]. It functions as a clathrin-uncoating ATPase in endocytosis [43,44] and aids in protein folding and degradation [45]. In contrast, *HSPA9*, which is predominantly located in the mitochondria, plays a crucial role in cell proliferation, stress response, and mitochondrial maintenance [46]. Previous studies have indicated that both *HSPA8* and *HSPA9* have been reported to significantly contribute to the prevention of apoptosis and oxidative stress [47–49]. In the present study, we found that the expression of *HSPA8* and *HSPA9* was significantly reduced in the serum of patients with sepsis-induced ALI compared to healthy individuals. ROS initiate a signaling cascade that activates the phosphorylation of the p66 Shc protein, subsequently enhancing the generation of hydrogen peroxide (H₂O₂). However, the heat shock protein HSPA8 can intervene in this process by inhibiting the p66 Shc signaling pathway, thereby preventing excessive production of ROS [50]. In contrast to previous studies, Tian et al. have found that *HSPA8* overexpression may decrease apoptosis and promoted the expression of the LPS-induced cytokines IL-1β, IL-6, and TNF-α in HD11 cells [51]. Additionally, superoxide dismutase 1 (SOD1), an antioxidant enzyme located in the cytosol and mitochondria, and it is associated with the levels of ROS [52,53]. Zhao et al. have showed that levels of *SOD1* mRNA is remarkably reduced in LPS-induced ALI compared to control group [54], which is consistent with our findings. These findings further support that *HSPA8*, *HSPA9* and *SOD1* might involve in the progression of sepsis-induced ALI via modulating the oxidative stress, apoptosis and inflammations. Thioredoxin (*TXN*) is a ubiquitous oxidoreductase regenerated from TXNRD2. Previous studies have indicated that *TXN* plays a significant role in the immune response and redox processes. It contributes to the defense against oxidative stress by regulating cellular free radicals and reactive oxygen species [55,56]. Although *TXN* exhibits significant upregulation in sepsis and its inhibition can reduce endoplasmic reticulum stress in cardiomyocytes [57], its primary function is localized intracellularly rather than being secreted into the bloodstream. Therefore, it is reasonable that we observed no significant difference in the expression levels of *TXN* in whole blood samples between sepsis-induced ALI group and the control group. α-Synuclein (*SNCA*) belongs to the synuclein family, it is closely associated with the etiology of neurodegenerative diseases [58], such as Alzheimer’s disease (AD) and Parkinsonism (PD). In contrast to previous studies, Tomlinson et al. have found that *SNCA*-null mice were less able to against infection after intravenous inoculation with Salmonella typhimurium in the bacterial sepsis model [59]. However, the role of SNCA in the lungs and other non-neural tissues remains incompletely understood. Our research demonstrated that there was no significant difference in the whole blood expression levels of *SNCA* between patients with sepsis-induced ALI and healthy subjects. These findings further support this observation may be attributed to the primary function of *SNCA* within the nervous system, suggesting that it does not directly participate in the mechanisms underlying sepsis-induced ALI, which accounts for the lack of significant variation in its whole blood expression levels.

In the present study, we discovered that the *VDAC1*, *HSPA8*, *HSPA9*, *SOD1*, *TXN*, and *SNCA* might involve in the oxidative stress related pathways, NF-κB signaling pathway and KEAP1-NRF2/NFE2L2 pathway. NF-κB serves as a crucial transcriptional regulator of inflammation-related genes. Its activation triggers the release of a cascade of inflammatory mediators that exacerbate sepsis [60]. In LPS-induced septic ALI/ARDS, pulmonary microvascular endothelial cells are stimulated to release TNF and IL-8, leading to increased intracellular calcium levels [61]. In a mouse model of sepsis-induced ALI, inhibiting the NF-κB signaling pathway significantly alleviated lung injury and improved survival rates [62]. Based on these observations, we hypothesized that these 6 oxidative stress-related genes might reduce inflammation by regulating the NF-κB pathway to prevent sepsis-induced ALI, which necessitates further experimental verification. In contrast to previous studies, the KEAP1-NRF2/NFE2L2 pathway is a crucial cellular signaling mechanism that regulates responses to oxidative stress, inflammation, and xenobiotic detoxification [63,64]. Activation of the Nrf2 signaling pathway has been reported to inhibit macrophage ferroptosis, thereby alleviating sepsis-induced ALI [65–68]. Uridine treatment has been shown to increase levels of Nrf2 and HO-1, decrease expression of KEAP1, improve survival rates, reduce pulmonary damage and bacterial presence, and modulate the Keap1-Nrf2 pathway [69]. Consistent with these results, we hypothesize that these six oxidative stress-related genes may regulate the progression of sepsis-induced ALI by modulating the KEAP1-NRF2/NFE2L2 pathway, which requires further experimental validation.

The proportion of neutrophils exhibited a significant negative correlation with the expression of *VDAC1*, *HSPA8*, *SOD1*, *HSPA9* and *TXN*, and a significant positive correlation with *SNCA* expression in sepsis-induced ALI. As immune effector cells, neutrophils play a pivotal role in the onset, progression and regression of various diseases, including ALI/ARDS [70]. These findings further support that in the early stages of sepsis, neutrophils are rapidly mobilized from the bone marrow into the blood circulation and recruited to the site of infection and injury to exert their phagocytic and bactericidal functions [71]. However, in the late stages of sepsis, neutrophils exhibit significant heterogeneity, dysfunction, immunosuppressive effects [72] and the release of neutrophil extracellular traps (NETs) [73]. In contrast to previous studies, these alterations may contribute to immune dysregulation and compromise the body’s ability to control infections. In sepsis-induced ALI/ARDS, neutrophils and NETs promote the pro-inflammatory and pro-angiogenic processes of endothelial cells, further impairing immune system function [61]. Based on these observations, considering the correlation between oxidative stress – related genes *VDAC1*, *HSPA8*, *SOD1*, *HSPA9*, *TXN*, *SNCA* and neutrophil proportion, we speculate that oxidative stress and neutrophils likely interact complexly in sepsis – induced ALI, affecting disease development and clinical outcomes. Given this interaction, targeting these mechanisms with therapies like NF - κB pathway or KEAP1-NRF2/NFE2L2 pathway inhibitors, antioxidants targeting the products of the identified genes, or small – molecule oxidative stress response element modulators might enhance the prognosis of sepsis patients.

The novelty of this study lies in its integration of multiple machine learning algorithms, which overcomes the limitations of conventional biomarker discovery methods by enhancing sensitivity, reducing false discovery rates, and better managing complex biological interactions. Through the integration of these algorithms, we successfully identified oxidative stress genes associated with sepsis-induced ALI and constructed an effective diagnostic model. This advancement not only enhances the understanding of sepsis-induced ALI but also provides new tools and strategies for the clinical diagnosis and treatment of this condition. Furthermore, the investigation of the correlation between oxidative stress-related genes and immune cells offers new insights into the role of immune cells in the pathogenesis of ALI. However, this study has several limitations. First, the samples utilized were sourced from public databases, which may introduce sample selection bias, potentially limiting their ability to adequately represent the broader patient population. To solve this, future studies should recruit more diverse patient groups in terms of ethnicity, age, comorbidities, and geography. This will boost the sample’s representativeness and the results’ external validity, making them applicable to a wider range of clinical settings. Second, the demographic and clinical information regarding sepsis-induced ALI was inadequately provided in the public datasets, limiting the study’s ability to reveal the clinical significance of the diagnostic genes within this patient population. Third, uncontrolled variables such as medication history, inflammatory status, and comorbid conditions may influence gene expression and should be considered in future validation studies. Lastly, the mechanisms by which the diagnostic genes influence the progression of sepsis-induced ALI require further investigation through *in vivo* experiments or clinical trials in future research.

## 5. Conclusion

In conclusion, by combining two machine learning algorithms with WGCNA analysis, this study has identified six oxidative stress-related genes in sepsis-induced ALI. These genes are associated with sepsis-induced ALI and may contribute to its pathophysiology, but further experimental validation is required. The logistic regression model developed using these six genes could identify patients with sepsis-induced ALI. Our findings have provided important insights into the molecular mechanisms underlying oxidative damage in sepsis-induced ALI and have helped develop novel research strategies to identify molecular therapeutic targets related to sepsis-induced ALI.

## Supporting information

S1 Fig Merging GSE32707 and GSE10361 datasets and removing batch effect. (A) The boxplot of the normalized data. Different colors represent different datasets. B. PCA results before batch removal for multiple datasets. C. PCA results after batch removal.
(TIF)

S2 Fig Determine the optimal soft threshold. According to the position of the red line, the soft threshold is set to 7 as the optimal choice for building a scale-free network.
(TIF)

S3 Fig Functional annotation analysis via Cytoscape and ClueGO Plug-in. A. KEGG pathways, B. biological processes (GO_BP). C. Cell components (GO_CC). D. molecular functions (GO_MF).
(TIF)

S4 Fig The results of LASSO regression algorithm. B. Ten cross-validations of the choice of adjustment parameters in the LASSO model. Each curve corresponds to one gene. A. LASSO coefficient analysis. Vertical dashed lines are plotted at the best lambda.
(TIF)

S5 Fig Merging GSE3037 and GSE172222 datasets and removing batch effect. A. The boxplot of the normalized data. Different colors represent different datasets. B. PCA results before batch removal for multiple datasets. C. PCA results after batch removal.
(TIF)

S1 Table Normal distribution results. (CSV)

S2 Table The genes related to sepsis-induced ALI onset in yellow and turquoise modules. (XLSX)

S3 Table Functional annotation analysis via Cytoscape and ClueGO Plug-in. (XLSX)

S4 Table The results of 145 sepsis-induced ALI-related oxidative stress genes. (XLSX)

S5 Table The results of GSVA and GSEA. (XLSX)

S6 Table The top 20 genes in the PPI network. (XLSX)

S7 Table Results of the logistic regression model. (XLSX)

S8 Table The model’s sensitivity, specificity, accuracy, positive predictive value, and negative predictive value. (XLSX)

S9 Table The result of pathway gene enrichment analysis was performed using the Reactome Pathway Database. (XLSX)

S10 Table The potential miRNA targeting *VDAC1*, *HSPA8*, *SOD1*, *HSPA9*, *TXN* and *SNCA.* (XLSX)
