# Supplementary figures and images for: Identification and validation of oxidative stress-related genes for the diagnosis of sepsis-induced acute lung injury
Source: PLoS One. 2025 Jul 22;20(7):e0327945. doi: 10.1371/journal.pone.0327945 (PMC12282930; doi:10.1371/journal.pone.0327945)

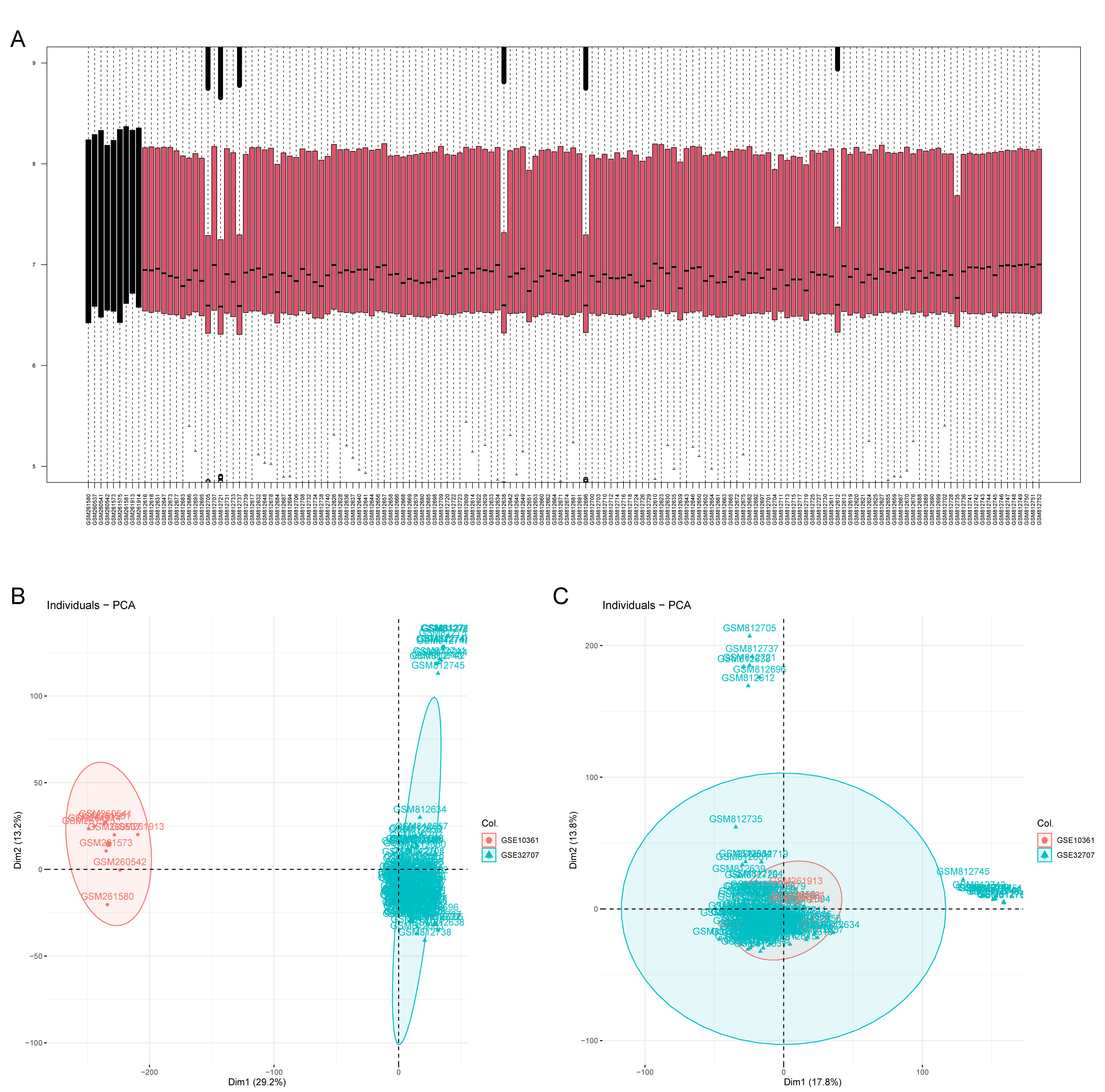

Supplement: S1 Fig — (A) The boxplot of the normalized data. Different colors represent different datasets. B. PCA results before batch removal for multiple datasets. C. PCA results after batch removal. (TIF) [file pone.0327945.s001.tif]

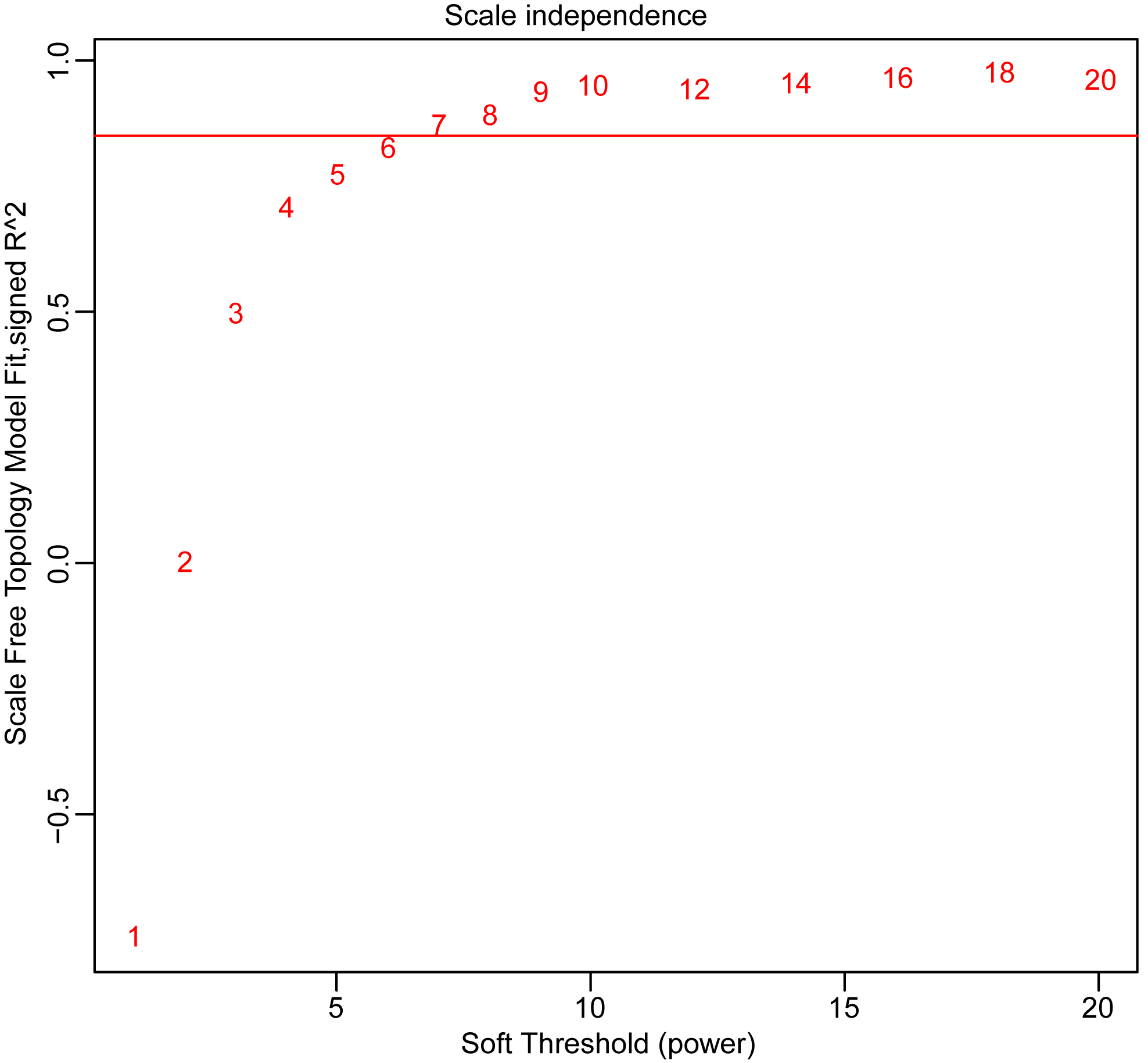

Supplement: S2 Fig — According to the position of the red line, the soft threshold is set to 7 as the optimal choice for building a scale-free network. (TIF) [file pone.0327945.s002.tif]

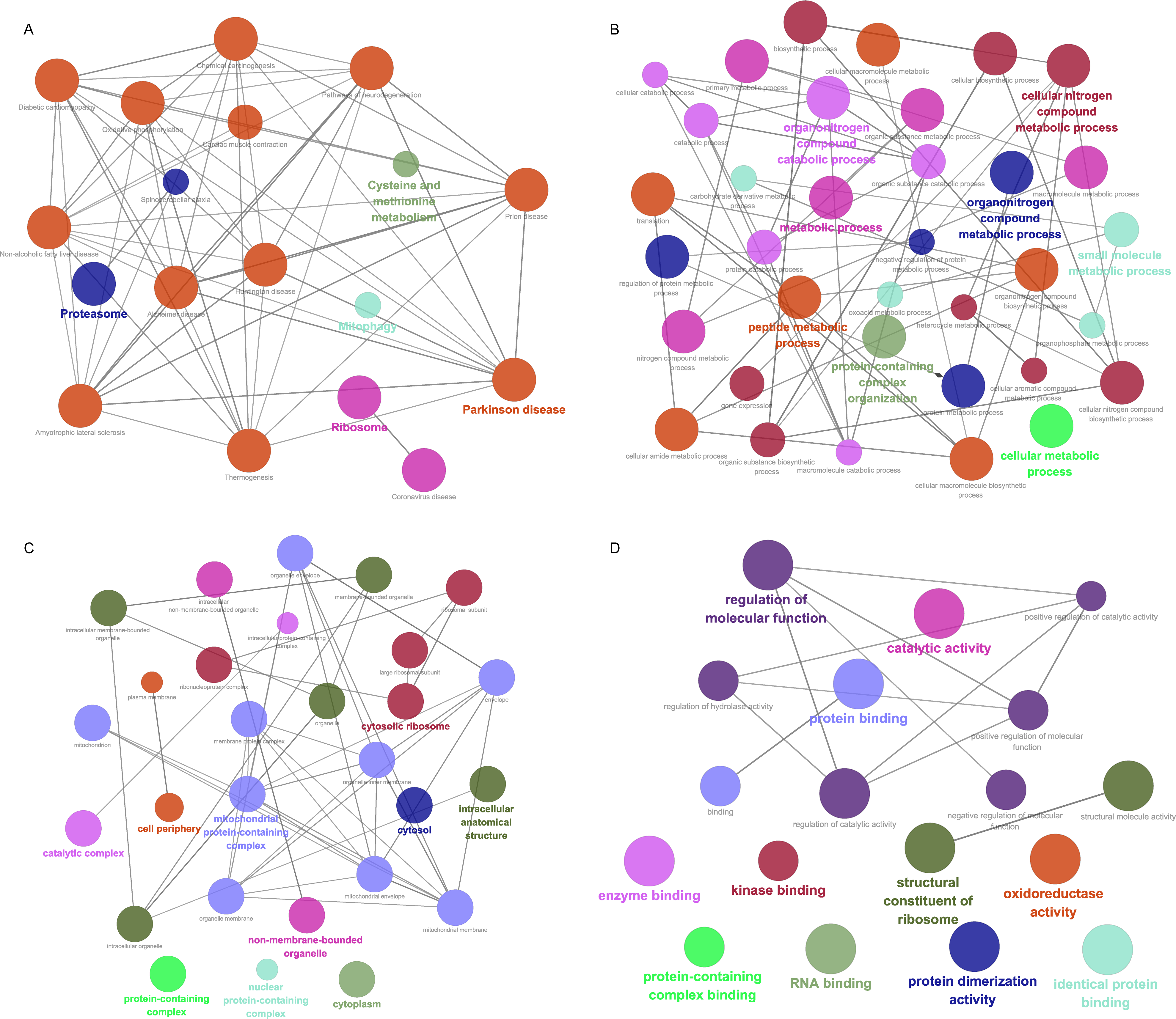

Supplement: S3 Fig — A. KEGG pathways, B. biological processes (GO_BP). C. Cell components (GO_CC). D. molecular functions (GO_MF). (TIF) [file pone.0327945.s003.tif]

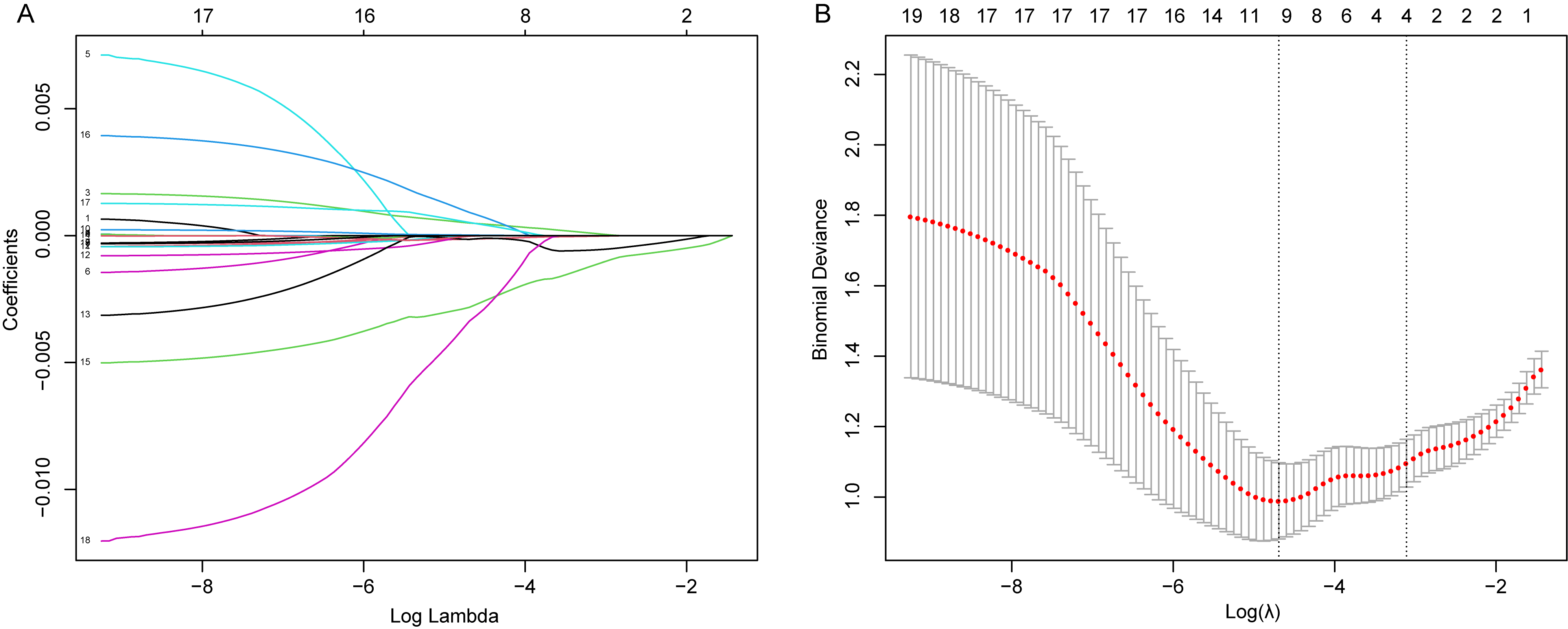

Supplement: S4 Fig — B. Ten cross-validations of the choice of adjustment parameters in the LASSO model. Each curve corresponds to one gene. A. LASSO coefficient analysis. Vertical dashed lines are plotted at the best lambda. (TIF) [file pone.0327945.s004.tif]

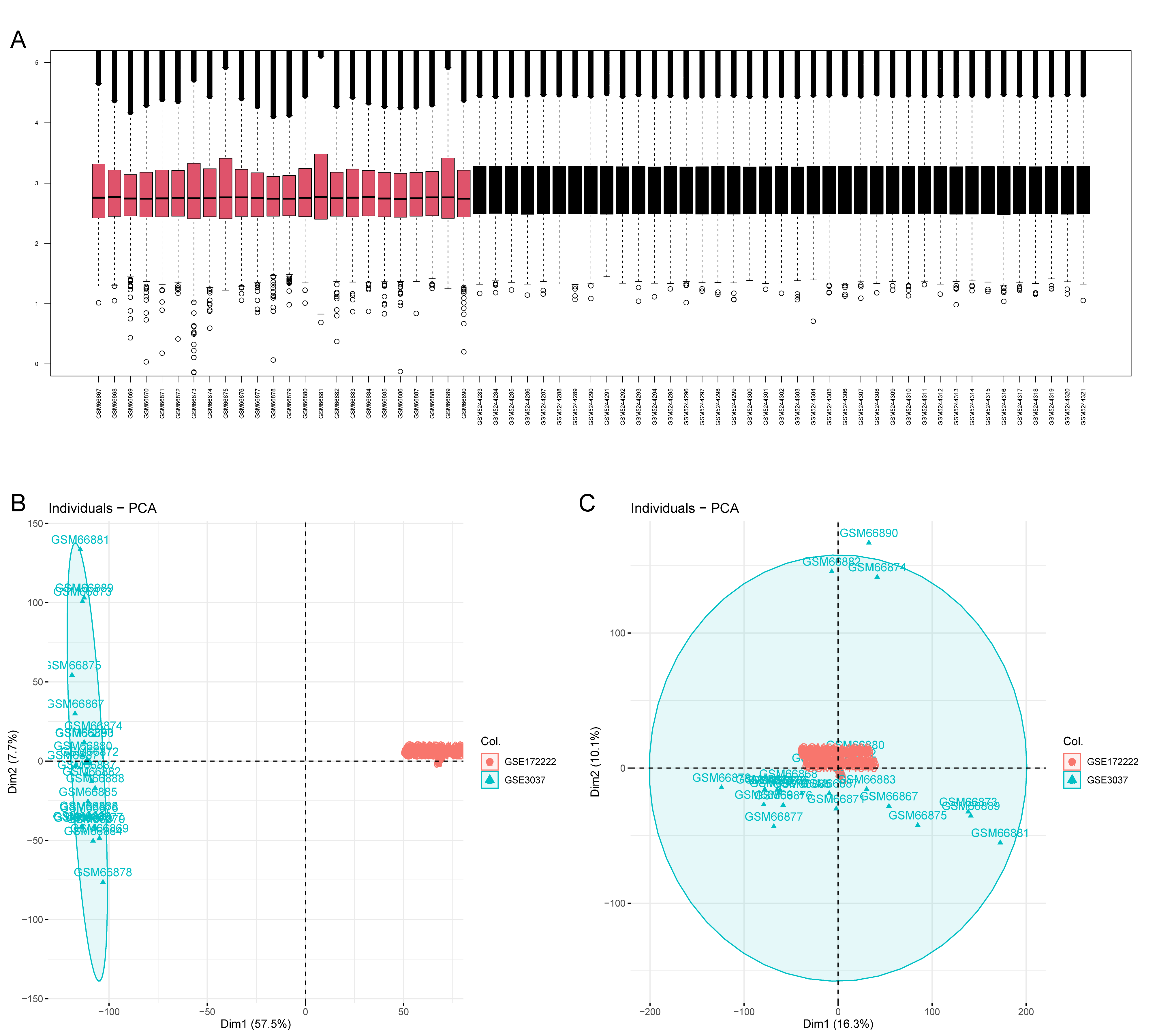

Supplement: S5 Fig — A. The boxplot of the normalized data. Different colors represent different datasets. B. PCA results before batch removal for multiple datasets. C. PCA results after batch removal. (TIF) [file pone.0327945.s005.tif]
